# Platelet-to-high-density lipoprotein ratio as a biomarker for asthma risk in adolescents: insights from NHANES 2011–2023

**DOI:** 10.3389/falgy.2025.1593351

**Published:** 2025-06-06

**Authors:** Fangju Liao, Qi Lv, Yana Xu, Jieyu Zan, Yu Xie

**Affiliations:** Putuo Hospital, Shanghai University of Traditional Chinese Medicine, Shanghai, China

**Keywords:** asthma, adolescents, platelet-to-HDL ratio (PHR), systemic inflammation, biomarker, NHANES

## Abstract

**Background:**

Asthma is a common inflammatory disease among adolescents, with systemic inflammation playing a key role in its development. The platelet-to-high-density lipoprotein ratio (PHR) has emerged as a novel indicator of systemic inflammation. However, while individual roles of platelet count and HDL cholesterol in asthma have been studied, the combined impact of PHR on asthma risk—particularly in adolescent populations—remains unclear and underexplored.

**Objective:**

This study aimed to evaluate the association between PHR and asthma risk in adolescents using data from the National Health and Nutrition Examination Survey (NHANES) 2011–2023.

**Methods:**

A cross-sectional analysis was conducted on 10,046 adolescents aged 8–18 years from NHANES. Asthma status was self-reported, and PHR was calculated as the ratio of platelet count (1,033 /μl) to HDL cholesterol (mg/dl). Logistic regression models were used to assess the association between PHR quartiles and asthma, adjusting for age, sex, race/ethnicity, body mass index (BMI), and poverty-to-income ratio (PIR). Restricted cubic spline (RCS) analysis was applied to examine non-linear relationships, with sensitivity and subgroup analyses conducted to ensure robustness.

**Results:**

Adolescents with asthma had higher PHR levels compared to non-asthmatic peers (*p* < 0.001). In fully adjusted models, participants in the highest PHR quartile had significantly higher odds of asthma (OR = 1.59, 95% CI: 1.37–1.83, *p* < 0.001) compared to the lowest quartile. RCS analysis revealed a non-linear relationship, with asthma risk escalating sharply at higher PHR levels. Subgroup analyses confirmed consistent associations across race/ethnicity, BMI, and PIR categories. Sensitivity analyses excluding participants under 12 years of age yielded similar results.

**Conclusion:**

PHR is significantly associated with asthma risk in adolescents, highlighting its potential as a cost-effective and accessible biomarker for identifying high-risk individuals. These findings underscore the need for further longitudinal and interventional studies to validate PHR’s role in asthma prediction and management.

## 1 Introduction

Asthma is a chronic inflammatory respiratory disease that significantly impacts adolescent populations, with studies reporting prevalence rates ranging from 10%–20% in many developed countries (1, 2). As adolescents experience unique physiological and immunological changes during puberty, they may be particularly vulnerable to inflammatory triggers that exacerbate asthma symptoms. Despite advances in asthma management, the upward trend in adolescent asthma prevalence underscores an urgent need to understand and mitigate potential risk factors, particularly those linked to systemic inflammation (3, 4). Recent research has identified inflammation as a critical component in asthma pathogenesis, driving interest in biomarkers that can reflect this inflammatory state and potentially predict asthma risk (5, 6). Furthermore, sex differences in asthma prevalence and severity become particularly pronounced around puberty, with males showing higher prevalence in childhood and females demonstrating increased rates post-puberty. These patterns suggest that hormonal and immunological shifts during adolescence may influence inflammatory responses differently across sexes (7).

The platelet-to-high-density lipoprotein cholesterol (HDL) ratio (PHR) has gained attention as a novel marker of systemic inflammation. Platelets, while primarily associated with hemostasis, play an active role in immune response and inflammation. Through the release of cytokines, interaction with leukocytes, and formation of platelet-leukocyte aggregates, platelets contribute to the inflammatory milieu that characterizes asthma exacerbations (8, 9). Elevated platelet counts have been linked to inflammatory diseases, including cardiovascular conditions and autoimmune disorders, suggesting a broader implication of platelet indices in chronic inflammatory diseases (10, 11).

Conversely, HDL cholesterol has anti-inflammatory and antioxidant properties, helping to counteract oxidative stress and neutralize inflammatory mediators (12, 13). Studies have demonstrated that reduced HDL levels are associated with increased inflammation, potentially exacerbating conditions like asthma. The PHR thus offers a combined perspective, integrating pro- and anti-inflammatory aspects into a single, accessible biomarker. Research into PHR has shown promise in predicting risks for various inflammatory and metabolic conditions, but studies specifically linking PHR to respiratory outcomes, particularly in adolescent populations, remain limited (14, 15). The inflammatory profile in asthma is typically dominated by Th2-driven responses, characterized by eosinophilic inflammation and elevated IL-4, IL-5, and IL-13. However, in some phenotypes, Th17 and mixed Th2/Th17 pathways may also contribute, particularly in severe or steroid-resistant asthma cases (16).

Several recent studies have examined inflammatory biomarkers in relation to asthma, focusing predominantly on adult populations or broader lipid-related indices rather than specific ratios such as PHR. For instance, research by Yao et al. (17) identified an association between low HDL levels and asthma severity in adults, while Kim et al. (18) noted increased platelet counts in asthmatic individuals, suggesting a role for platelet indices in asthma pathophysiology. However, these studies did not examine the combined effect of platelet and HDL levels, leaving a gap in understanding how the interplay of these two parameters might influence asthma risk, particularly in adolescents.

Furthermore, adolescent studies on asthma biomarkers have typically focused on more traditional inflammatory markers such as C-reactive protein (CRP) or eosinophil counts, with limited consideration of lipid-based ratios or indices. While CRP has been extensively studied, it lacks specificity as an asthma predictor, as elevated CRP levels are associated with a wide range of inflammatory conditions (19, 20). The innovation of the present study lies in the use of PHR as a novel, integrated marker that reflects both pro-inflammatory platelet activity and the anti-inflammatory role of HDL. By focusing on adolescents, this study provides valuable insights into how systemic inflammation may influence asthma susceptibility during a critical period of physical and immunological development (21, 22).

Our study utilizes data from the National Health and Nutrition Examination Survey (NHANES) 2011–2023 dataset, one of the most comprehensive sources for examining health-related trends across diverse populations. Leveraging NHANES data, we aim to assess the association between PHR and asthma risk specifically in adolescents, providing an evidence-based evaluation of PHR as a potential biomarker for identifying high-risk individuals. If validated, PHR could offer a simple, cost-effective marker for early asthma risk assessment, guiding preventive interventions in a demographic where early management is critical to improving long-term outcomes.

## 2 Methods

### 2.1 Study design and population

**Figure 1:**
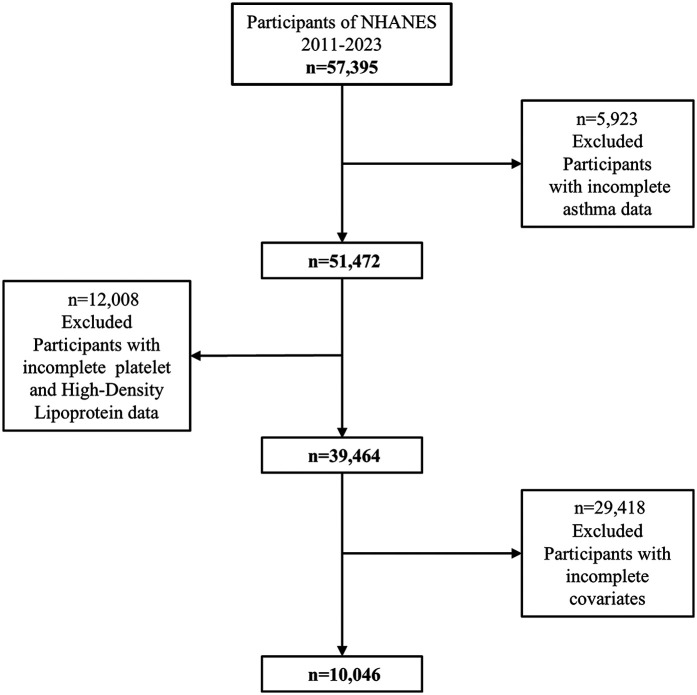
Flow chart of participant selection.

This study analyzed data from the National Health and Nutrition Examination Survey (NHANES) collected between 2011 and 2023, employing a cross-sectional design. The initial dataset included 51,472 participants of all ages. Participants with missing asthma data (*n* = 11,008) were excluded, resulting in 40,464 individuals. After further excluding those with incomplete information on HDL cholesterol or platelet count (*n* = 12,008) and missing covariates (*n* = 6,920), a total of 39,464 participants remained. To focus on adolescents, the analysis was restricted to individuals aged 8–18 years, leaving 10,046 participants in the final sample. The detailed participant selection process is illustrated in Figure 1. Participants with known major comorbidities such as diabetes, cancer, cardiovascular disease, or chronic kidney disease were excluded based on self-reported medical conditions and examination files, to minimize confounding effects.

### 2.2 Outcome and exposure definitions

Asthma status was determined based on self-reported responses to the question, “Has a doctor or other health professional ever told you that you have asthma?” Participants were classified as having asthma or not based on this binary variable (23). The primary exposure, platelet-to-HDL ratio (PHR), was calculated by dividing platelet count (measured in 1,033 /μl) by HDL cholesterol level (measured in mg/dl) (8). PHR was analyzed both as a continuous variable and as a categorical variable, divided into quartiles (Q1–Q4) based on the distribution within the study population.

### 2.3 Covariates

The analysis included several covariates identified from the literature as potential confounders. These included age, sex, race/ethnicity (Mexican American, Other Hispanic, Non-Hispanic Black, Non-Hispanic White, and Other Races), body mass index (BMI; BMI categories (underweight, normal weight, overweight, obese) were classified using CDC growth chart percentiles for age and sex(Supplementary Figures S1, S2): underweight (<5th percentile), healthy weight (5th–85th percentile), overweight (85th–95th percentile), and obesity (≥95th percentile), and poverty-to-income ratio (PIR; ≤1, 1–3, >3). All covariates were included in the multivariate models to adjust for their potential effects on the relationship between PHR and asthma.

### 2.4 Statistical analysis

Baseline characteristics were summarized separately for participants with and without asthma. Continuous variables were compared using unpaired (independent) t-tests, as the compared groups (asthmatic vs. non-asthmatic) were mutually exclusive and unrelated, while categorical variables were analyzed using chi-square tests. Weighted logistic regression was performed to assess the association between PHR and asthma, accounting for the complex survey design of NHANES. Three models were constructed: an unadjusted model, a model adjusted for age and sex, and a fully adjusted model incorporating all covariates. Trend analysis was conducted by treating PHR quartiles as an ordinal variable.

To explore non-linear associations, restricted cubic spline regression was applied. Subgroup analyses were conducted to evaluate potential effect modifications by race/ethnicity, BMI, and PIR, with formal tests for interaction. Sensitivity analyses were performed by excluding participants under 12 years of age to confirm the robustness of the findings. All statistical analyses accounted for the NHANES sampling weights and were conducted using appropriate survey-based methods.

### 2.5 Ethical considerations

NHANES was approved by the National Center for Health Statistics Research Ethics Review Board, and informed consent was obtained from all participants or their legal guardians. As this study used publicly available de-identified data, additional ethical approval was not required.

## 3 Results

### 3.1 Baseline characteristics of the study population

The study included a total of 10,046 participants aged 8–18 years, of whom 2,037 (20.3%) were identified as having asthma. The baseline characteristics of the study population stratified by asthma status are shown in Table 1. Participants with asthma were slightly younger than those without asthma (11.87 ± 3.72 vs. 12.20 ± 3.58 years, *p* < 0.001). There was a significant difference in the gender distribution, with a higher proportion of females in the asthma group (55.7% vs. 49.9%, *p* = 0.014). Racial composition also varied, with a higher prevalence of asthma observed among Non-Hispanic Black participants (5.5% vs. 22.7%, *p* = 0.004).

**Table 1:** Baseline characteristics of the study participants.

Characteristics	Overall	Asthma		*P*-value
		No	Yes	
n	10,046	8,009	2,037	
Age, years	11.94 ± 3.69	12.20 ± 3.58	11.87 ± 3.72	<0.001
Gender, *n* (%)				0.014
Female	5,128(%)	3,993(%)	1,135(%)	
Male	4,918(%)	4,016(%)	902(%)	
Race, *n* (%)				0.004
Mexican American	2,028 (20.2%)	1,689 (16.8%)	339 (3.4%)	
Other Hispanic	1,178 (11.7%)	902 (9.0%)	276 (2.7%)	
Non-Hispanic Black	2,833 (28.2%)	2,280 (22.7%)	553 (5.5%)	
Non-Hispanic White	2,361 (23.5%)	1,779 (17.7%)	582 (5.8%)	
Other races	1,646 (16.4%)	1,359 (13.5%)	287 (2.9%)	
PIR, *n* (%)				0.005
≤1	3,049 (30.3%)	2,379 (23.7%)	670 (6.7%)	
1–3	4,290 (42.7%)	3,429 (34.1%)	861 (8.6%)	
>3	2,707 (26.9%)	2,201 (21.9%)	506 (5.0%)	
BMI				<0.001
Underweight	3,773 (37.6%)	714 (7.1%)	3,059 (30.5%)	
Healthy Weight	4,404 (43.8%)	827 (8.2%)	3,577 (35.6%)	
Overweight	1,161 (11.5%)	295 (2.9%)	866 (8.6%)	
Obesity	698 (7.0%)	201 (2.0%)	497 (5.0%)	
HDL-cholesterol (mg/dl)	53.65 ± 12.31	54.04 ± 12.26	52.08 ± 12.40	0.264
Platelet count (1,000 cells/ul)	275.58 ± 61.97	273.52 ± 60.52	283.71 ± 66.76	0.001

BMI, body mass index; PIR, the ratio of family income to poverty.

Socioeconomic status, as indicated by the poverty-to-income ratio (PIR), was associated with asthma prevalence. Participants with asthma were more likely to belong to lower PIR categories (*p* = 0.005). Additionally, BMI categories differed significantly between the asthma and non-asthma groups (*p* < 0.001), with higher rates of overweight and obesity observed in the asthma group. No significant differences were found for HDL cholesterol levels between the two groups (*p* = 0.264), while platelet counts were significantly higher in participants with asthma (283.71 ± 66.76 vs. 273.52 ± 60.52 × 1,033 /μl, *p* = 0.001).

### 3.2 Association between platelet-to-HDL ratio and asthma

Weighted logistic regression analyses evaluating the association between PHR and asthma are presented in Table 2. In the unadjusted model (Model 1), participants in the highest PHR quartile (Q4) had significantly higher odds of asthma compared to those in the lowest quartile (Q1) (OR = 1.73, 95% CI: 1.51–1.98, *p* < 0.001). After adjusting for age and sex (Model 2), the association remained significant (OR = 1.81, 95% CI: 1.58–2.07, *p* < 0.001). The fully adjusted model (Model 3), which accounted for age, sex, race/ethnicity, BMI, and PIR, showed a consistent positive association between higher PHR and asthma (OR = 1.59, 95% CI: 1.37–1.83, *p* < 0.001). Trend analysis revealed a statistically significant dose-response relationship across PHR quartiles in all models (p for trend <0.001).

**Table 2:** Weighted logistic regression analyses of association between PHR and asthma.

Categorizations	Model 1		Model 2		Model 3	
	OR 95%CI	*P* value	OR 95%CI	*P* value	OR 95%CI	*P* value
Q1	Reference		Reference		Reference	
Q2	0.96 (0.83, 1.11)	0.581	0.98 (0.85, 1.14)	0.801	0.95 (0.82, 1.10)	0.522
Q3	1.15 (0.99, 1.32)	0.059	1.18 (1.03, 1.36)	0.022	1.10 (0.95, 1.28)	0.186
Q4	1.73 (1.51, 1.98)	<0.001	1.81 (1.58, 2.07)	<0.001	1.59 (1.37, 1.83)	<0.001
P for trend		<0.001		<0.001		<0.001

Model 1: no covariates were adjusted.

Model 2: age and sex were adjusted.

Model 3: age, sex, race, BMI, PIR were adjusted.

95% CI, 95% confidence interval.

### 3.3 Restricted cubic spline (RCS) analysis

**Figure 2:**
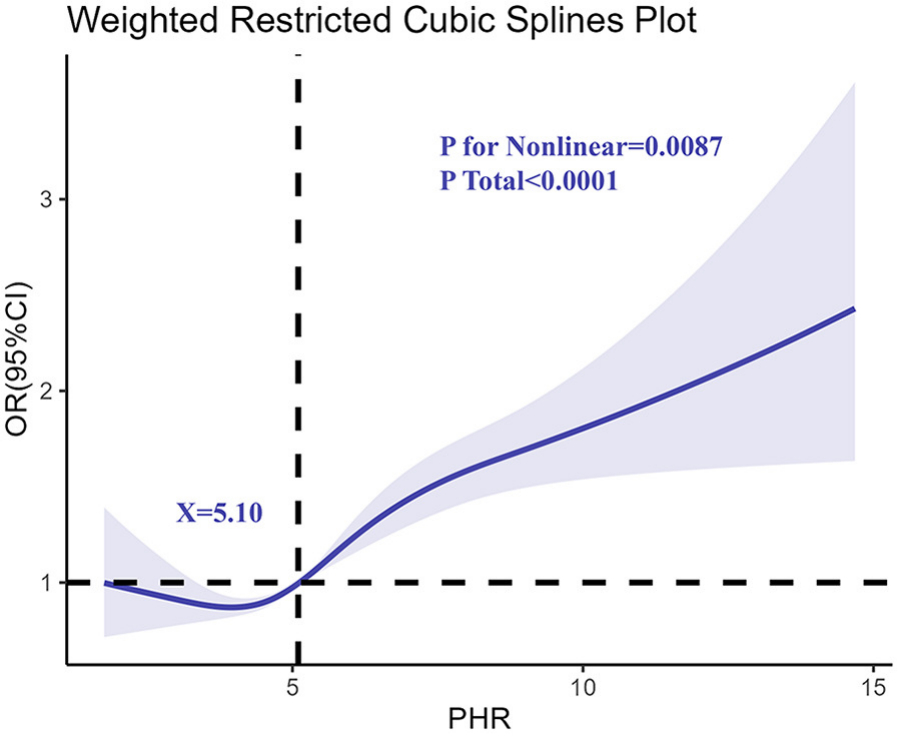
Determination of the association between PHR and adolescents asthma by restricted cubic spline (RCS) regression analysis.

To explore the potential non-linear relationship between PHR and asthma risk, restricted cubic spline regression was applied (Figure 2). The analysis confirmed a non-linear association, with asthma risk increasing sharply at higher PHR levels. This pattern was consistent after adjusting for age, sex, race/ethnicity, BMI, and PIR, suggesting that the risk of asthma escalates more rapidly as PHR levels surpass a certain threshold. The spline curve further emphasized the robustness of PHR as a predictive biomarker for asthma.

### 3.4 Subgroup and interaction analyses

**Figure 3:**
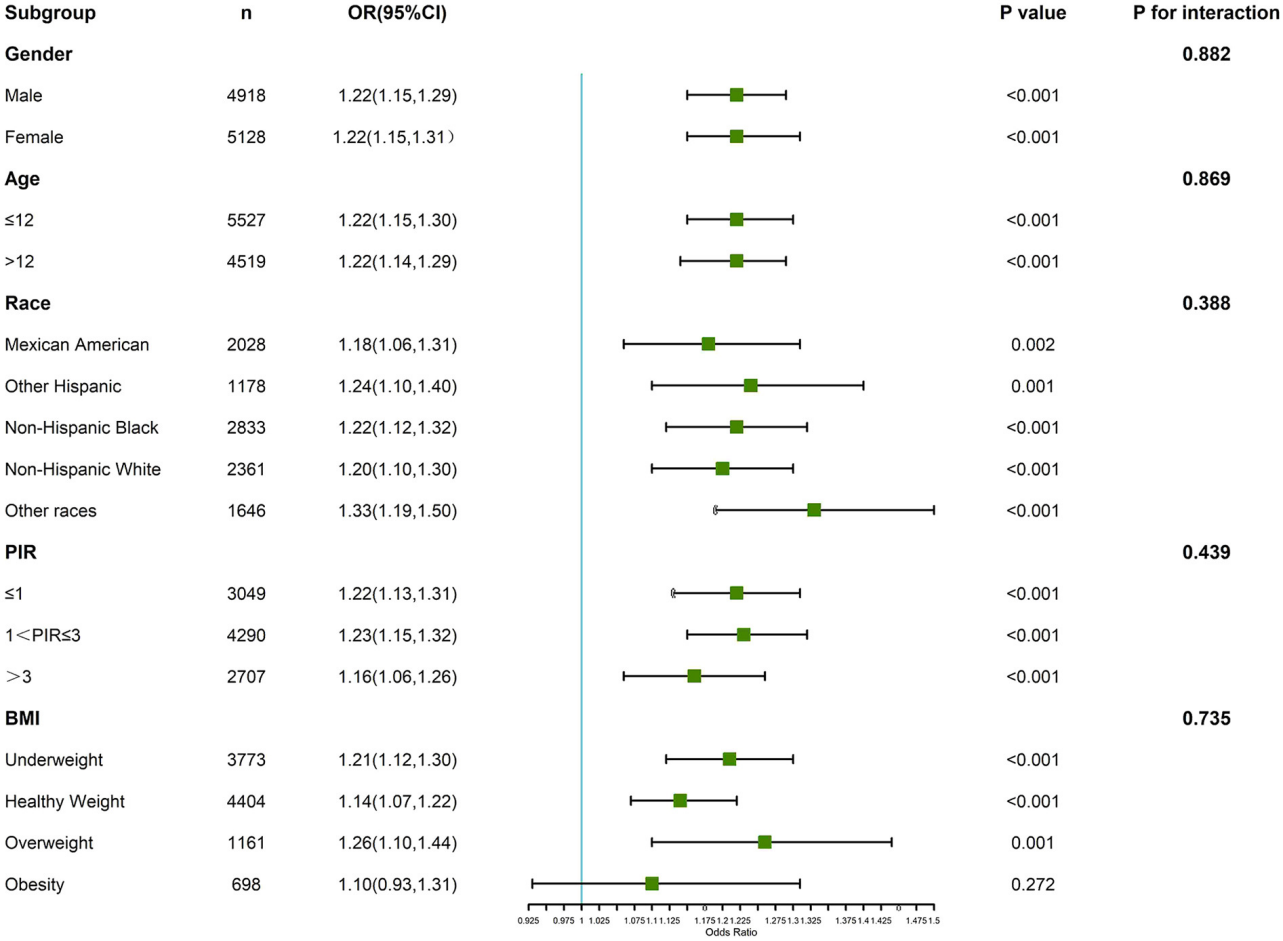
Verification of the association between PHR and adolescents’ asthma by subgroup analyses and interaction analyses.

Subgroup analyses were conducted to examine the association between PHR and asthma across various demographic and clinical categories, including race/ethnicity, BMI, and PIR (Figure 3). The association between higher PHR and asthma was consistent across most subgroups, with participants in the highest PHR quartile showing increased odds of asthma compared to those in the lowest quartile. While the strength of the association varied slightly, it was more pronounced in Non-Hispanic Black participants and individuals with higher BMI categories. Interaction analyses revealed no statistically significant interactions between PHR and these covariates, indicating that the relationship between PHR and asthma was broadly similar across these subgroups.

### 3.5 Sensitivity analysis

After excluding participants younger than 12 years, the association between PHR and asthma was re-evaluated (Table 3). The results were consistent with the primary analysis, with the highest quartile of PHR (Q4) showing a significant association with asthma in the fully adjusted model (OR = 1.58, 95% CI: 1.27–1.96, *p* < 0.001). The dose-response relationship remained robust, with p for trend <0.001 in all models.

**Table 3:** The Sensitivity analysis of the association between PHR and asthma after excluding individuals under 12 years of age.

Categorizations	Model 1		Model 2		Model 3	
	OR 95%CI	*P* value	OR 95%CI	*P* value	OR 95%CI	*P* value
Q1	Reference		Reference		Reference	
Q2	0.89 (0.71,1.11)	0.291	0.92 (0.73,1.15)	0.472	0.91 (0.73,1.14)	0.418
Q3	1.02 (0.82,1.27)	0.862	1.08 (0.86,1.34)	0.516	1.03 (0.82,1.29)	0.797
Q4	1.64 (1.33,2.01)	<0.001	1.76 (1.43,2.16)	<0.001	1.58 (1.27,1.96)	<0.001
P for trend		<0.001		<0.001		<0.001

Model 1: no covariates were adjusted.

Model 2: age and sex were adjusted.

Model 3: age, sex, race, BMI, PIR were adjusted.

95% CI, 95% confidence interval.

## 4 Discussion

This study highlights the significant association between platelet-to-HDL ratio (PHR) and asthma risk in adolescents, adding to the growing body of evidence on the role of systemic inflammation in chronic respiratory diseases. Adolescents with higher PHR, especially those in the highest quartile, demonstrated a disproportionately elevated risk of asthma. These findings support the hypothesis that PHR, as a composite marker reflecting both pro-inflammatory platelet activity and anti-inflammatory HDL function, can provide a nuanced assessment of systemic inflammatory status and its relationship with asthma.

Although several biomarkers such as CRP, eosinophil counts, and fractional exhaled nitric oxide (FeNO) have been used to assess asthma-related inflammation, each has notable limitations. CRP lacks specificity, eosinophil counts vary by phenotype, and FeNO primarily reflects airway eosinophilia without capturing systemic inflammation. Emerging candidates like periostin, YKL-40, and IL-17 have shown promise, but they are either costly, less accessible, or limited to specific asthma endotypes. In this context, PHR provides an integrative, readily available marker that captures both pro- and anti-inflammatory components of systemic inflammation (24, 25).

The results align with and extend prior studies that have examined the individual components of PHR. Elevated platelet counts have been implicated in asthma exacerbations, likely due to their role in amplifying inflammation via cytokine release, chemotaxis, and the formation of platelet-leukocyte aggregates (26–28). Conversely, reduced HDL cholesterol, with its antioxidant and anti-inflammatory properties, has been associated with increased systemic inflammation and oxidative stress, factors known to worsen asthma outcomes (29–31). While previous research has predominantly focused on these components in isolation, our study integrates them into a single metric, demonstrating that PHR may be more predictive of asthma risk than either marker alone. Notably, traditional biomarkers such as C-reactive protein (CRP) or eosinophil counts, while useful, often lack specificity to asthma and are influenced by a wide range of inflammatory conditions (32, 33). PHR addresses this limitation by combining two biologically relevant markers, capturing a broader spectrum of inflammatory dynamics.

The restricted cubic spline analysis provides critical insights into the non-linear nature of the PHR-asthma relationship. This finding suggests that while mild to moderate elevations in PHR may not substantially increase asthma risk, levels beyond a certain threshold are associated with a sharp rise in risk. This pattern aligns with the “inflammatory threshold hypothesis,” which posits that compensatory mechanisms can mitigate mild systemic inflammation, but once these mechanisms are overwhelmed, disease processes such as airway hyperresponsiveness and chronic inflammation ensue (34–36). These observations are consistent with findings from cardiovascular and metabolic research, where high PHR has been linked to increased risks of chronic inflammatory diseases, underscoring its role as a systemic marker of heightened inflammatory burden (8, 37).

Comparing our findings with existing literature also underscores potential racial and ethnic disparities in asthma risk. Subgroup analyses revealed a particularly strong association between PHR and asthma in Non-Hispanic Black adolescents, a group already known to have a higher prevalence of asthma and greater exposure to environmental and socioeconomic risk factors (6, 38). Additionally, the association between PHR and asthma was more pronounced in individuals with higher BMI, suggesting that obesity-related inflammation may amplify the systemic inflammatory processes captured by PHR. These observations support calls for tailored public health interventions targeting vulnerable subgroups to mitigate asthma risk.

The potential mechanisms underlying the PHR-asthma relationship are grounded in the interplay between platelets and HDL in modulating inflammation. Platelets, traditionally associated with thrombosis, are now recognized as key players in immune regulation and inflammatory signaling. Their activation can trigger the release of pro-inflammatory cytokines, promote leukocyte recruitment, and enhance endothelial dysfunction, all of which contribute to airway inflammation and hyperresponsiveness (39, 40). HDL, on the other hand, acts as a counter-regulatory agent, neutralizing inflammatory mediators, reducing oxidative stress, and improving endothelial function (41, 42). A high PHR, therefore, reflects a state of unopposed pro-inflammatory activity, potentially exacerbating asthma-related pathophysiological changes. Experimental studies have shown that disrupting the platelet-HDL balance in animal models can worsen respiratory outcomes, further supporting the biological plausibility of our findings (43, 44).

From a public health perspective, the identification of PHR as a novel biomarker for asthma risk has several implications. PHR is a simple, cost-effective, and widely available metric derived from routine blood tests, making it particularly suitable for large-scale screening and risk stratification in clinical and community settings. By identifying adolescents at elevated risk of asthma, PHR could enable earlier intervention strategies, including lifestyle modifications, targeted pharmacotherapy, and enhanced monitoring to prevent disease progression (21, 45). Furthermore, integrating PHR into clinical practice could complement existing diagnostic and prognostic tools, improving the precision of asthma management while reducing healthcare disparities (46).

Despite its strengths, this study has limitations that warrant consideration. The cross-sectional design precludes causal inferences, as it is unclear whether elevated PHR precedes asthma development or is a consequence of chronic inflammation. Future longitudinal studies are needed to establish temporal relationships and causal pathways (47). Additionally, asthma diagnosis was based on self-reported data, which, while practical for large-scale surveys, may be prone to recall bias or misclassification. Although we adjusted for key confounders, residual confounding cannot be entirely ruled out. Finally, the findings are specific to U.S. adolescents and may not be generalizable to other populations or age groups (48).

Future research should focus on validating these findings in longitudinal cohorts and exploring the utility of PHR as a predictive biomarker for asthma exacerbations and other inflammatory conditions. Mechanistic studies investigating the pathways linking PHR to airway inflammation and immune dysregulation would further elucidate its role in asthma pathogenesis (49). Additionally, clinical trials evaluating the impact of interventions aimed at reducing PHR, such as anti-inflammatory therapies or HDL-enhancing strategies, could provide valuable insights into its therapeutic potential. Expanding these efforts to include diverse populations, including adults and high-risk subgroups, would further strengthen the evidence base for PHR as a versatile biomarker (50). Participants under 12 years of age were excluded in sensitivity analyses to address potential misclassification of asthma diagnosis in younger children, who may have transient wheezing or viral-induced respiratory symptoms not representative of chronic asthma.

In conclusion, this study identifies PHR as a novel and promising biomarker for asthma risk in adolescents, reflecting the balance between pro-inflammatory and anti-inflammatory forces. By capturing systemic inflammatory dynamics, PHR offers a unique opportunity to enhance early detection, guide personalized interventions, and ultimately improve outcomes for individuals at risk of asthma. These findings represent an important step toward integrating inflammatory biomarkers into routine asthma care and prevention strategies.

## Data Availability

The original contributions presented in the study are included in the article/Supplementary Material, further inquiries can be directed to the corresponding author.
