# Supplementary figures and images for: Platelet-to-high-density lipoprotein ratio as a biomarker for asthma risk in adolescents: insights from NHANES 2011–2023
Source: Front Allergy. 2025 Jun 6;6:1593351. doi: 10.3389/falgy.2025.1593351 (PMC12179139; doi:10.3389/falgy.2025.1593351)

**
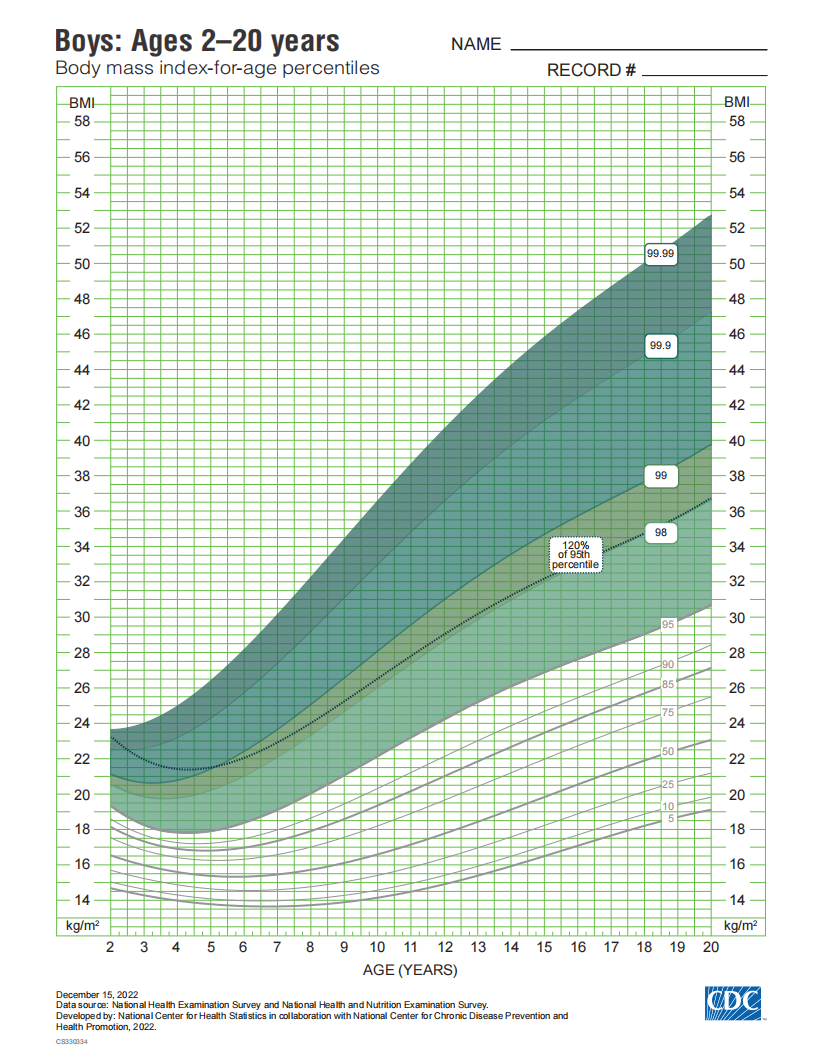
**

**Figure S1.** CDC growth chart -boy


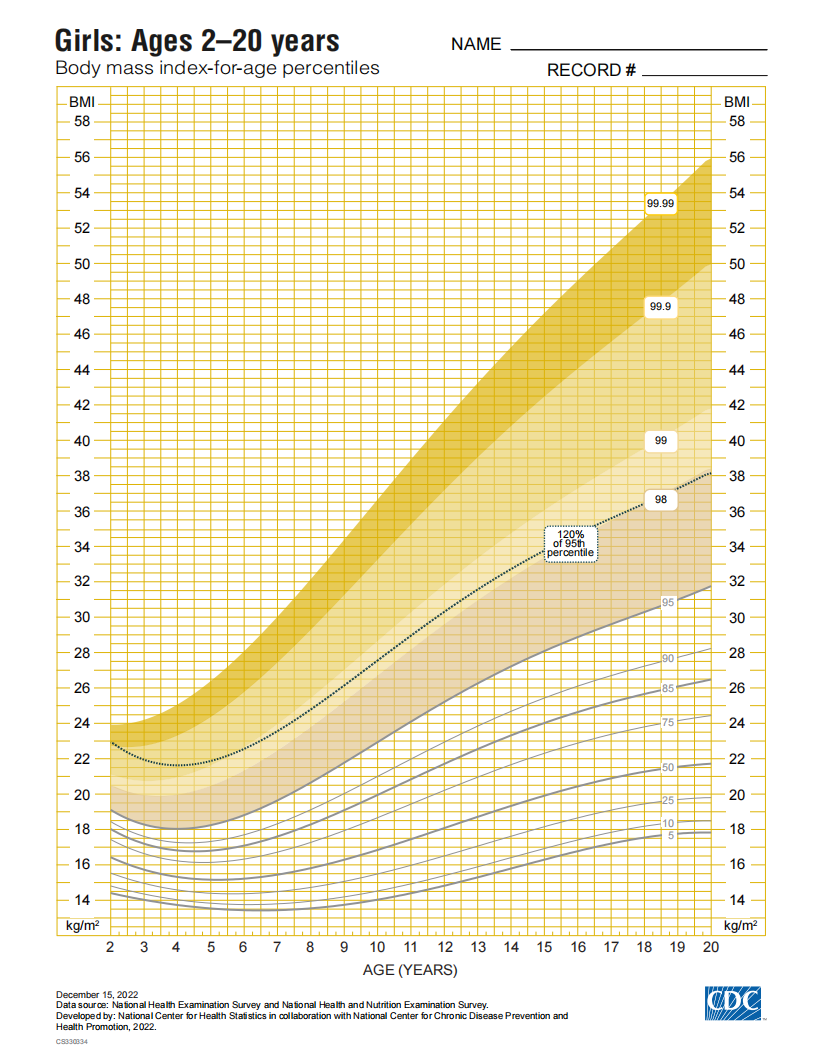


Figure S2. CDC growth chart -girl

Supplement: Supplementary file 1 [file Datasheet1.docx]
